# Silicon‐Embedded Multifunctional Heterogeneous Integration for Miniaturized Photoplethysmography Detection Devices

**DOI:** 10.1002/advs.75416

**Published:** 2026-04-28

**Authors:** Lang Chen, Shizun Hu, Zhou Yang, Shitao Shen, Pan Zhang, Han Xu, Chi Zhang, Wei Wang

**Affiliations:** ^1^ School of Integrated Circuits Peking University Beijing China; ^2^ National Key Laboratory of Advanced Micro and Nano Manufacture Technology Beijing China; ^3^ Beijing Advanced Innovation Center for Integrated Circuits Beijing China

**Keywords:** heterogeneous integration, multifunctional device, thermal management, wearable electronics

## Abstract

The multifunctional integration of chips with high flexibility and scalable manufacturing is crucial for enhancing chip performance, reducing chip size, and simplifying chip design. However, balancing volume, cost, flexibility, and functionality using traditional heterogeneous integration methods is challenging. To overcome this, a silicon‐embedded multifunctional heterogeneous integration method based on top‐down engineering was proposed. As a representative application, a wearable photoplethysmography detection system was demonstrated. The functional modules of this system required for sensing, acquisition, and processing were embedded and reconnected on the same silicon interposer to form a new integrated chip. A thermal‐aware floorplan optimization algorithm was employed to improve the thermal performance of the integrated chip, achieving a 6.5°C reduction in the peak temperature. The chip accurately detected physiological changes across different frequency ranges following physical activity performed at varying intensities by test participants. Compared to traditional integration methods, the proposed method achieved a 46% and 94.7% reduction in power consumption and volume, respectively, and a remarkable 90% increase in functional unit density. This technology promotes rapid, flexible, and low‐cost manufacturing of multifunctional chips toward the development of next‐generation multifunctional, low‐power, miniaturized electronic devices.

## 1 Introduction

The rapid expansion and diversification of application scenarios has accelerated the evolution of electronic devices with multifunctional and highly integrated configurations [1, 2, 3]. Wearable electronics, as an emerging application scenario for the miniaturization of electronic systems, impose stringent requirements on volume, device density, power consumption, and thermal stability [4, 5, 6]. Printed circuit boards have been previously employed to integrate packaged chips into “sensor–process–communication” systems [6, 7]. Despite their simplicity, the bulky form, density limitations, and high static power consumption of these boards cause hinderance in achieving the miniaturization and energy‐efficiency demands of advanced integrated electronic systems [7].

With breakthroughs in semiconductor technology, heterogeneous integration (HI) has emerged as a transformative solution, enabling high‐density integration of different components, including sensors, analog circuits, and digital chips, manufactured using different substrates and process nodes to form an integrated chip with complete “sensor–process” functionality [8, 9, 10, 11]. However, the monolithic integration of heterogeneous chips fabricated on dissimilar substrates and process nodes remain technically challenging [12]. Chip‐level HI facilitates the cross‐platform integration of intellectual property from different chip design entities [13, 14, 15], overcoming the limitations of monolithic single‐chip, single‐silicon designs [16]. Moreover, this enables the implementation of complex system‐on‐chip (SoC) functions on a single silicon wafer, which would be impractical if these functions were confined to a single chip. Modular design methodologies allow the use of verified chip modules in rapid prototyping, accelerating development [11, 17]. This approach decouples the integration process from lengthy redesign cycles associated with full‐custom SoC development [18], significantly shortening product launch times. By avoiding the expensive fully custom design and manufacturing processes inherent in traditional single‐chip SoC development, the chip‐based integration method, which leverages chip modules, reduces development costs by several orders of magnitude.

In chip‐level multifunction integration, packaging technology provides a platform for efficient collaboration between components in a unified package [19]. Current multi‐chip‐module technologies suffer from limited integration density and significant signal propagation delays [20], and the chip‐on‐wafer‐on‐substrate and embedded multi‐die interconnect bridge technologies are constrained by microbump bonding‐induced input/output scalability limitations [21, 22, 23]. Integrated fan‐out, although structurally simplified through face‐up chip integration, faces thermal management bottlenecks due to epoxy substrates [24]. Moreover, the rising demand for higher density and performance in HI exacerbates thermal management challenges because of the differences in power dissipation and thermal sensitivity across heterogeneously integrated chips [25]. Therefore, developing integration technologies that can achieve high‐density integration for various chip types, strong thermal dissipation, and low power consumption to support HI is a critical priority.

This study proposes a silicon‐embedded multifunctional heterogeneous integration (SEMHI) approach that can significantly reduce chip size, power consumption, and manufacturing costs. Given that SEMHI technology offers significant advantages in chip‐level HI, it is well suited for application in photoplethysmography (PPG)‐based diagnosis. PPG detection is a critical application in wearable electronics, where miniaturization, low power consumption, and thermal efficiency are essential [26, 27, 28]. Traditional integrated devices are bulky and consume high power, particularly when used in wearable or implantable forms. Therefore, SEMHI holds significant application potential and practical value.

In the SEMHI method, the PPG signal‐detection system was first disintegrated into independent functional chips, each designed to perform specific tasks, such as signal sensing, acquisition, and processing. Then, using a multi‐objective optimization algorithm driven by a simulated annealing algorithm, an optimal layout that minimized both volume and thermal performance was built. The results of functional testing confirmed that the integrated system meets the required detection functions, validating the potential of this modular, reconfigurable approach in achieving low‐power, high‐performance integration. The proposed method not only addresses critical challenges in thermal management and system miniaturization but also offers a scalable, flexible platform for the development of multifunctional devices. Its application to PPG signal detection sets a precedent for advancements in low‐power, low‐working‐temperature, and miniaturized electronic systems.

## 2 Results

### 2.1 Overview

**FIGURE 1:**
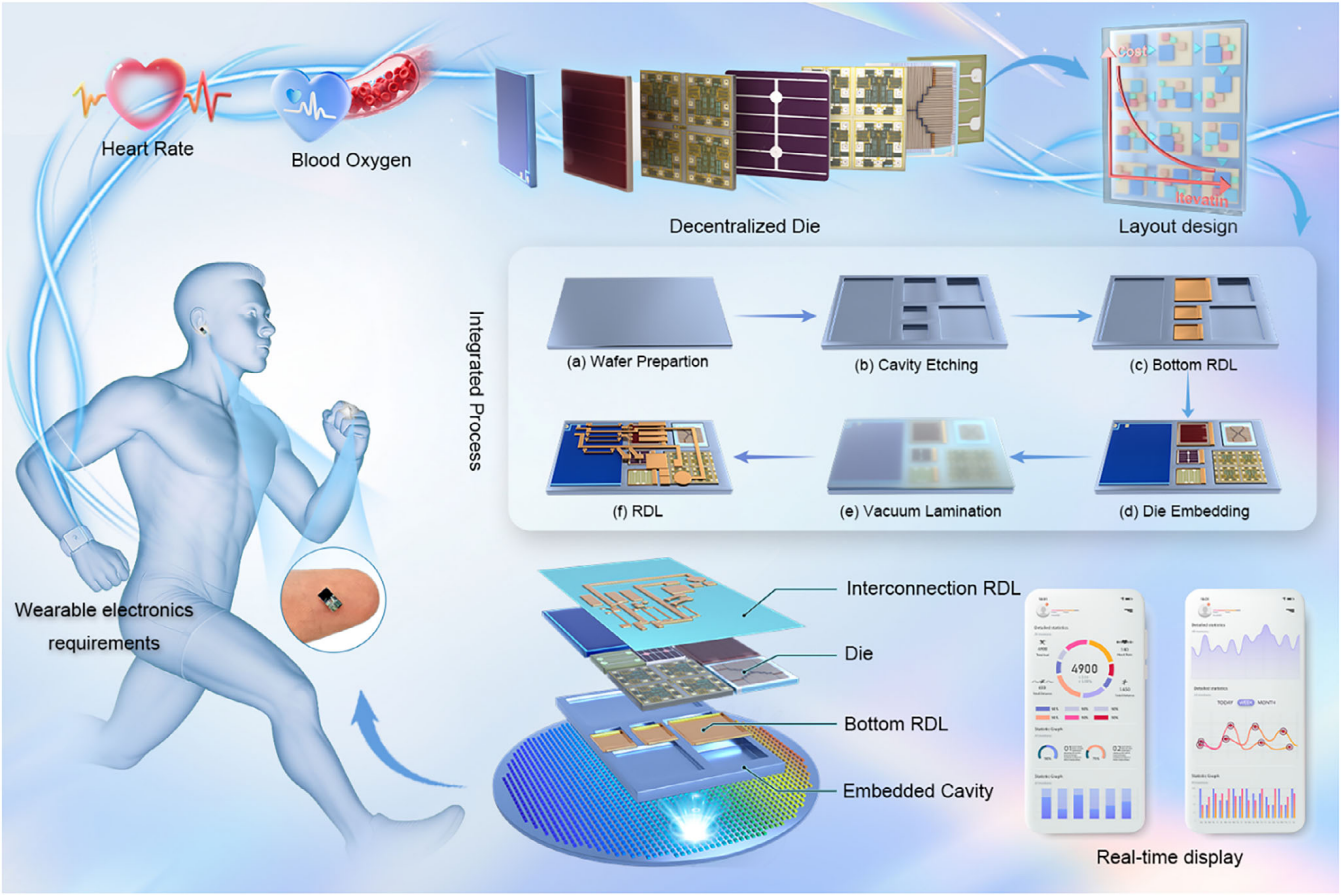
Schematic of PPG detection devices.

Figure 1 presents a schematic of the integrated PPG detection devices that were developed following several important steps. First, the functional modules of the target chip were analyzed, and discrete functional chips that could satisfy target signal sensing, acquisition, and processing requirements were selected. Next, the key parameters of the functional chips (such as their size and thermal power) were extracted as inputs, and a thermal layout optimization model algorithm was developed to perform layout optimization, yielding the optimal layout of the chip under the constraints of key parameters such as temperature, area, and line length. Finally, based on the layout results, the corresponding layout was designed. Using the embedded reconfigurable technology on silicon, the functional chips were integrated into a single chip with multiple functions.

### 2.2 Floorplan Optimization

To verify the effectiveness of the floorplan optimization method for HI, three chip layouts for different optimization targets were built and evaluated through experiments. The three layout designs were created through volume optimization (VO), temperature optimization (TO), and volume–temperature optimization (VTO). VO mainly optimized the volume and wire length of the chips, with a volume cost to wire length cost ratio of approximately 1:1. TO optimized the volume, wire length, and temperature of chips, with a focus on temperature. The ratio of the volume, wire length, transient temperature, and steady‐state temperature costs was approximately 1:1:9:9. TO was achieved by increasing the proportion of temperature terms in the total cost. VTO involved comprehensive optimization of the chip volume, wire length, transient temperature, and steady‐state temperature with a cost ratio of approximately 1:1:1:1. The initial temperature of annealing was 100°C, the final temperature was 0.01°C, and the cooling rate was 0.996.

**FIGURE 2:**
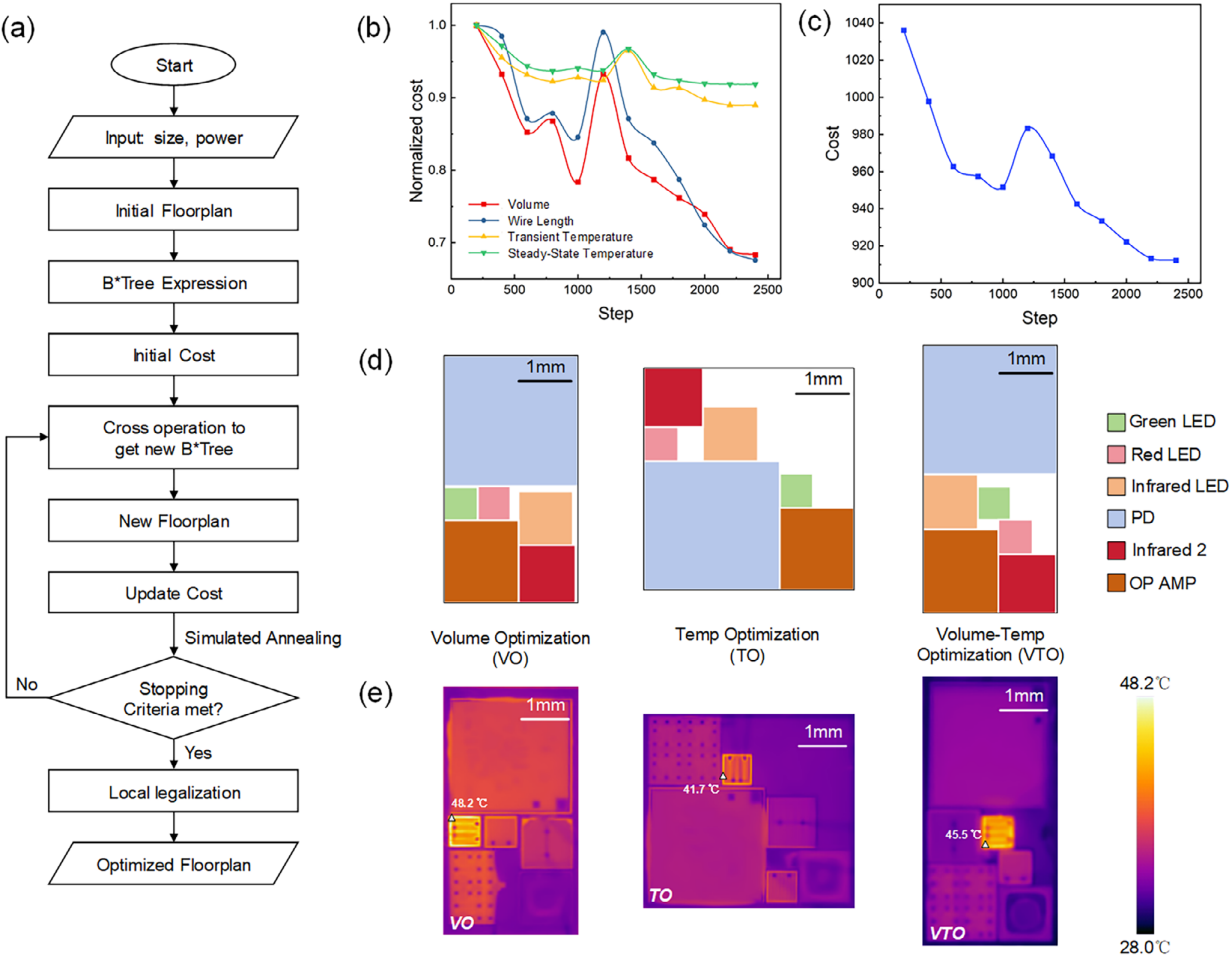
Floorplan optimization of integration: (a) flowchart of floorplan optimization based on B^*^Tree and the simulated‐annealing algorithm; (b) cost convergence process of the volume, wire length, and transient and steady‐state temperature; (c) convergence process of the total cost in simulated annealing; (d) optimized floorplans for three different designs; (e) thermal infrared images of the three optimized designs.

To clearly demonstrate the optimization process of simulated annealing, we normalized each cost item. In each iteration, the ratio of the new cost value to the initial cost value was calculated, i.e., *Normalized* *cost*  = *cost_new_*/*cost_initial_* , where *cost* refers to α · *Area*, β · *WL*, γ · *T_transient_*, and ε · *T_stable_*, and *cost_initial_* is the initial cost value of the first manual floorplan. Figure 2b shows the reduction process of the four cost items during the optimization iteration. Compared to the temperature terms, the reductions in volume and wire length costs were more significant. In addition, as the chip shrinks in volume, the wiring length tends to decrease, meanwhile the temperature tends to increase as the distance between the hotspots decreases. Therefore, the volume and wire length costs exhibited similar trends, which were opposite to that of the temperature cost. However, each cost item generally exhibited a fluctuating downward trend. This is because, in the process of volume reduction, the floorplan optimization algorithm provides a floorplan where hotspots are as far apart as possible under the same volume to achieve the optimal solution among volume, wire length, and temperature. Figure 2c presents the reduction process of the total cost, the overall trend of which is downward with a significant reduction range. As the optimization iteration proceeds, the cost curve may increase locally. This is because the simulated‐annealing algorithm randomly accepts the worst solution according to the Boltzmann probability function, thereby escaping the local optimum solution, which is the main feature distinguishing the simulated‐annealing algorithm from the greedy algorithm [29].

Figure 2d presents the layout results of the three designs and the corresponding results of different floorplans, respectively. The numerical results can be found in Table 1. VO only optimizes the volume and wire length; thus, its floorplan is the most compact, but the temperature is relatively high. In TO, a longer distance between chips is beneficial for reducing the temperature; thus, the floorplan is relatively scattered, and the temperature is the lowest. VTO involves comprehensive target optimization, including the volume, wire length, and temperature; thus, all the indicators are between those of VO and TO. To further verify the effectiveness of this method, we prepared thermal testing samples based on the three optimization outcomes. We performed temperature tests at room temperature (approximately 20°C), using an infrared (IR) temperature detector to measure the chip temperature. Figure 2e presents the IR temperature distributions of the actual chips in the three designs, all of which operated under direct‐current conditions. Compared with TO, VO featured a more compact chip arrangement, which led to more pronounced heat accumulation and a higher maximum temperature. The peak temperature of VO was 6.5°C higher than that of TO. Additionally, while VO and VTO had comparable volumes, VTO achieved superior temperature performance owing to its optimized layout.

**TABLE 1:** Inputs and results for different optimization targets.

Optimization target		Volume	Temp.	Volume & temp.
Inputs	α (mm^−2^)	2.5	2.5	2.5
	β (mm^−1^)	1	1	1
	γ (°C^−1^)	0	9	0.8
	ε (°C^−1^)	0	10	0.9
	*T_initial_*	100	100	100
	*T_end_*	0.01	0.01	0.01
	Cooling rate	0.996	0.996	0.996
Outputs	Volume (mm^3^)	6.7	9.1	7.2
	Wire length (mm)	29.9	47.3	33
	Steady‐state temp. (°C)	49.1	44.4	47.9
	Transient temp. (°C)	39.7	38.5	39.2

### 2.3 Silicon‐Embedded Integration

**FIGURE 3:**
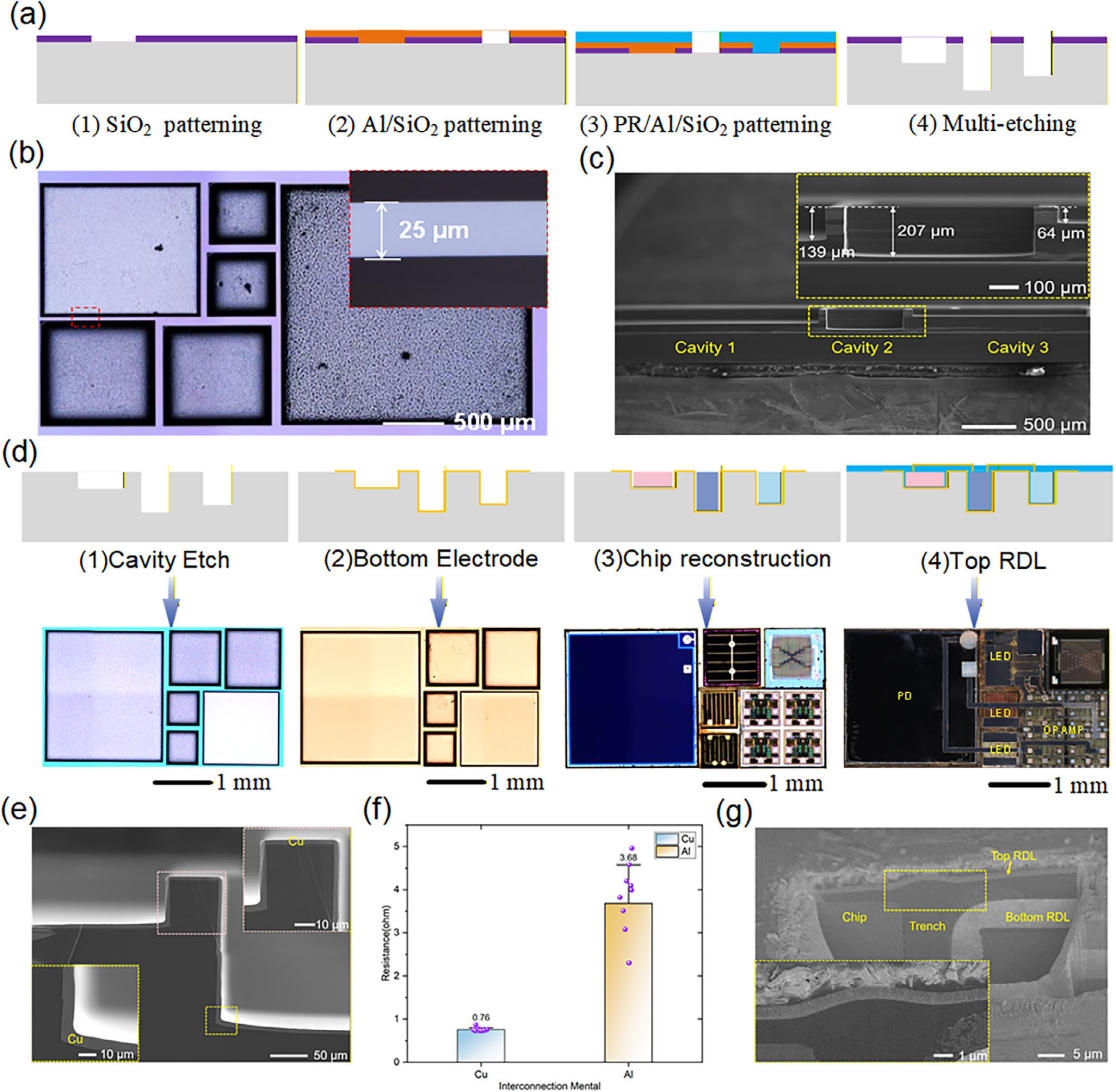
Fabrication results of the SEMHI method. (a) Cross‐sectional diagram of the multiscale embedded cavity fabrication process flow; (b) minimum spacing between embedded cavities; (c) depth test results of the fabricated embedded cavities; (d) cross‐section flowchart and related physical diagrams of the key wafer reconstruction processes; (e) bottom and sidewall morphologies of the metallized embedded cavities; (f) resistance measurements of copper and aluminum base electrodes; (g) morphology of electrical interconnections in failure‐prone areas such as trenches.

As shown in Figure 3b, the minimum spacing between embedding cavities was <25 µm, which is significant for reducing chip interconnect distances and increasing chip integration density. As shown in Figure 3c, a maximum etching‐depth difference of 143 µm was observed between the cavities for chip embedding. The actual etching depth of the embedding cavities deviated from the expected etching depth by less than ±3 µm. Furthermore, owing to the reduced surface undulations, higher precision was achieved in the patterning of the embedding trenches. Figure 3e illustrates the cavity after the metallization process. The copper layer grown on the bottom and sidewalls of the embedding cavities exhibited a consistent thickness across different regions, resulting in a well‐formed copper interconnect structure with no cracks or substrate detachment. Excellent coverage was achieved even in failure‐prone volumes such as pattern corners. Additionally, we tested the electrical performance of the interconnect structure from the cavity bottom to the surface using different metallic materials, and the results are shown in Figure 3f. In probe station testing, the copper interconnect structure exhibited a resistance of only 0.76 Ω, whereas aluminum with equivalent physical dimensions registered 3.68 Ω, which is 4.8 times higher. This disparity primarily stems from copper's lower electrical resistivity and the greater ease in achieving substantial thickness through electroplating.

The integrated‐chip layout generated by the layout algorithm was subjected to the corresponding manufacturing processes, as illustrated in Figure 3a,d. First, multilayer thin‐film processes were used to etch various embedding‐cavity layouts. The etching error of the embedded cavities was <2 µm, and the depths met the design expectations. No surface deformation or bottom‐film overflow was observed. Next, the bottom electrodes were fabricated at the bottom of the embedded cavities for chips requiring bottom‐electrode connections. As shown in Figure 3e, the bottom electrodes provide excellent coverage at the trench bottom, allowing the electrodes to extend from the bottom to the surface through the sidewalls, which facilitated subsequent chip interconnection and testing.

Subsequently, multiple chips were embedded into their respective cavities, the detailed parameters of the chips can be found in Table S2. After embedding, the chip surface morphology remained intact, with no cracking at the bottom or die‐attach film (DAF) overflow. A dry‐film vacuum lamination process was used to fill the gap between the embedded chips and the silicon interposer layer. The cross‐section of the filled structure is presented in Figure 3f. As shown, the dry film effectively fills deep trenches (21.5 µm wide, with an aspect ratio exceeding 6) without forming keyhole effects. This step is crucial for enhancing the reliability of integrated chips. For trenches with smaller apertures and higher aspect ratios, vacuum‐deposited Parylene can be considered for filling [30]. Following surface passivation, the chip interconnect redistribution layer (RDL) was fabricated.

Prior to the RDL fabrication, the chip interconnect pads were exposed. Because the chips may not be perfectly centered after embedding, it is critical to control embedding errors for achieving high‐density interconnects in an integrated chip. Therefore, the reserved gap width was reduced to control the chip displacement within the trenches, increasing the interconnection density. According to prior research [30], the minimum achievable gap width is 5 µm. The pads were then subjected to photolithography. Testing of individual pad displacement revealed that photolithographic deviation during interconnect processes after packaging was controlled within ±5 µm. This tolerance range meets the requirements of most chip interconnect applications. Finally, RDL patterns were formed by sputtering a 1 µm thick aluminum interconnect metal layer onto the surface and performing patterning. As shown in Figure 3g, cross‐sectional scanning electron microscopy (SEM) observations of the RDL revealed well‐defined connections even in critical regions such as wire cross‐trenches.

### 2.4 Function Test of Integrated Chip

**FIGURE 4:**
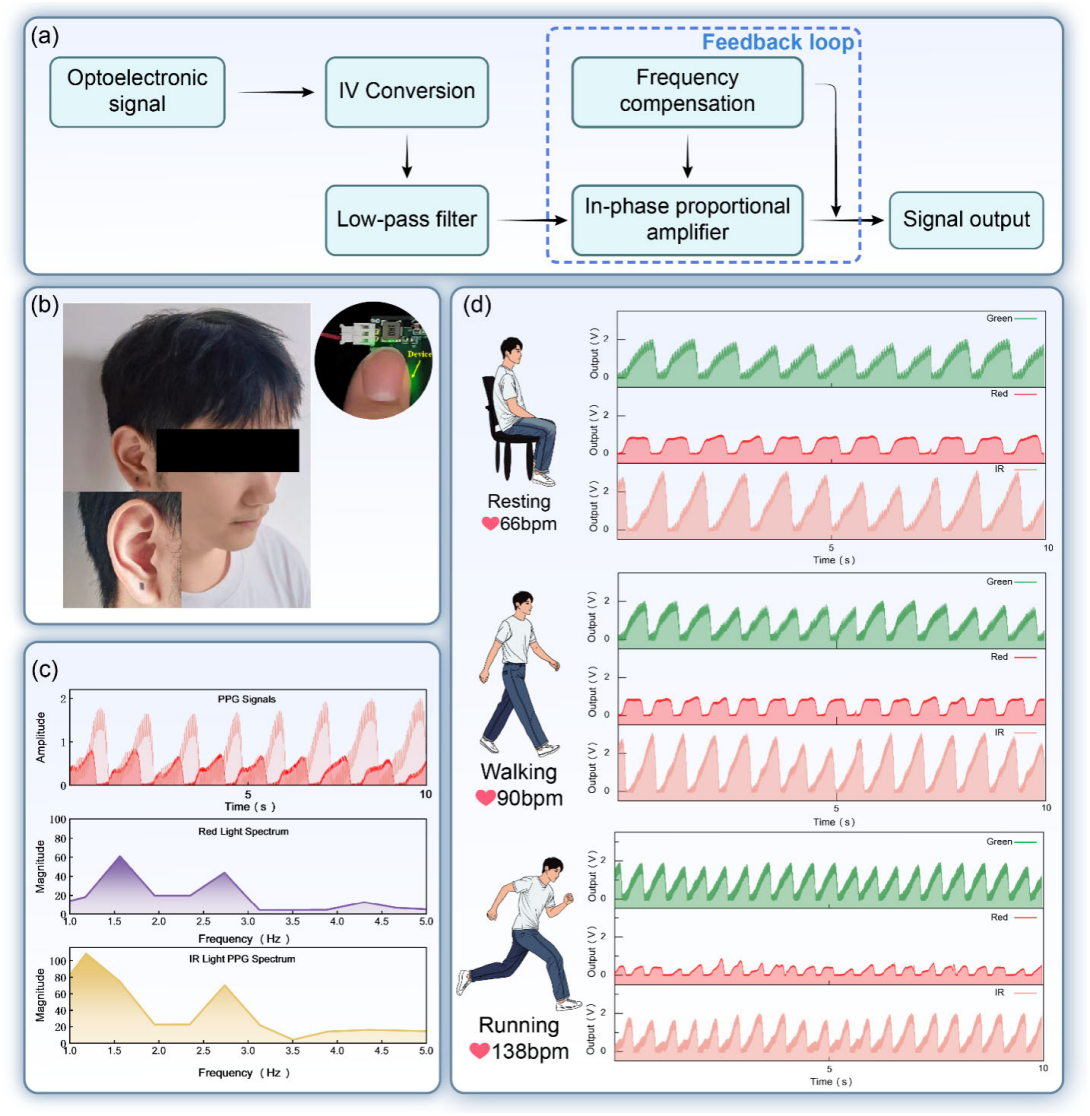
Functional testing of integrated chips (VTO). (a) Recognizable PPG signals obtained after processing raw photometric signals. (b) Chip worn on the earlobe as a wearable device. (c) Calculation of blood oxygen using the PPG method. (d) Dynamic PPG signal testing in different human activity states.

This integrated chip incorporates multiple‐wavelength light‐emitting diodes (LEDs), photodetector chips, and operational‐amplifier integrated circuits (ICs), enabling the initial gain amplification of the optical signals directly within the chip. To convert the dynamic optical signals captured by the chip into recognizable waveforms, the signals undergo the processing steps outlined in Figure 4a. First, the captured signal undergoes current‐to‐voltage (IV) conversion. Because the chip is designed specifically for human signal acquisition with a sampling frequency below 1 kHz, the converted waveform is subjected to low‐pass filtering to eliminate high‐frequency interference. A feedback loop consisting of a non‐inverting amplifier and a frequency compensator amplifies and outputs the signal. To transmit the processed signal, corresponding board‐level circuits were designed to control the signal acquisition and wireless transmission.

Figure 4b shows the integrated chip worn on the human body for PPG signal measurement. When worn on the body, the chip acquires voltage–time waveforms from key skin areas, such as the fingertips and ears, from which the current heart‐rate information is extracted. Additionally, by exploiting the difference in absorption ratios between oxygenated and deoxygenated hemoglobin at different wavelengths, the corresponding blood‐oxygen information can be extracted from the waveform. A dual‐wavelength PPG algorithm using red and IR light is employed to calculate blood oxygen saturation. The signal‐processing steps for this are illustrated in Figure 4c. First, the raw signal is transformed into the frequency domain using a fast Fourier transform (FFT). The spectral ratio *R* is defined as the amplitude ratio between the dominant frequency components of red and IR light. The calculated *R* value is substituted into empirical Equation (1) [31] to determine the blood oxygen concentration at this point. As shown in Figure 4c, the calculated blood‐oxygen concentration was approximately 97.5%.

(1) $$\;SpO_{2}=-45.06*R^{2}+30.354*R+94.845$$

Finally, the algorithm was programmed and integrated into the microcontroller unit (MCU) of the control board. Combined with a wireless Bluetooth transmission module, this allowed continuous monitoring of physiological parameters during various states of human activity. As shown in Figure 4d, the experimental design required the subjects to perform a graded exercise protocol that included resting, walking, and running. The chip continuously acquired real‐time PPG signals through onboard algorithms, simultaneously outputting heart rate and blood oxygen saturation data. This information was then transmitted to the host display via a Bluetooth module. The integrated chip reliably and stably acquires PPG signals at different frequencies following physical activity of varying intensities, confirming its excellent detection capabilities and operational reliability.

## 3 Discussion

**FIGURE 5:**
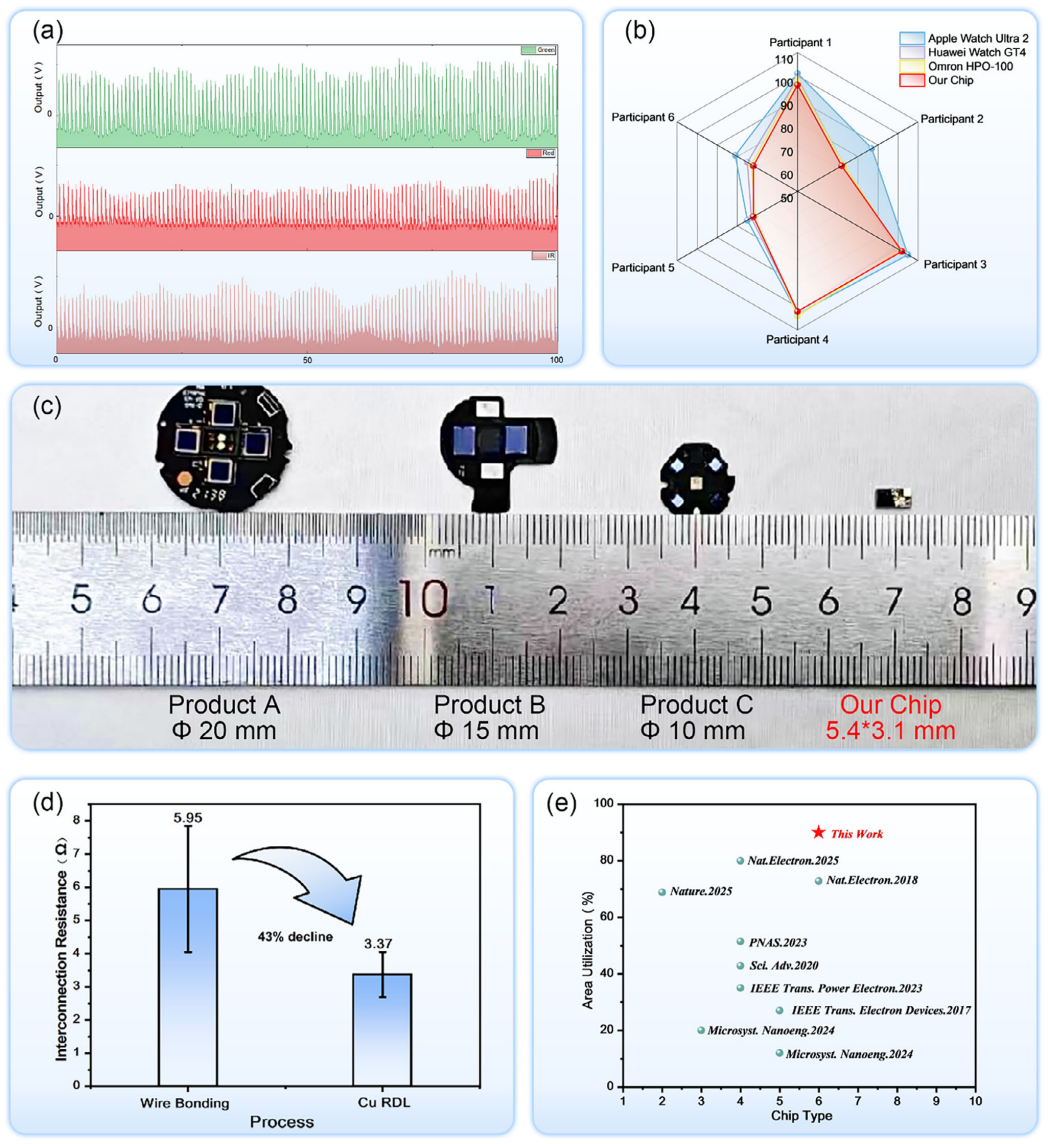
Performance of SEMHI. (a) Long‐term (0–100 s) monitoring of the chip; (b) comparison with other commercial products in heart‐rate detection; (c) volume comparison with commercial products; (d) power consumption reduced by 43% compared to traditional interconnection methods; (e) comparison between previously reported results and the chip fabricated in this study in terms of integrated functional unit density [11, 17, 32, 33, 34, 35, 36, 37, 38].

Figure 5a presents continuous PPG waveforms acquired by the proposed chip over a 100 s duration. The graph illustrates synchronized hemodynamic signals detected under green, red, and IR illumination. Throughout this extended recording period, the device exhibited exceptional temporal stability and signal integrity. Notably, all three spectral channels displayed high‐fidelity waveforms characterized by distinct systolic peaks and diastolic troughs. Furthermore, the waveforms exhibited negligible baseline drift and consistent pulsatile amplitude, indicating the chip's robust resistance to thermal drift and electronic noise accumulation. The cycle‐to‐cycle consistency observed over the 100 s window confirms the sensor's excellent repeatability. Collectively, these results validate the reliability of the proposed chip for long‐term, continuous physiological monitoring in wearable health applications.

Figure 5b presents a comparative analysis of heart‐rate detection accuracy between the proposed photonic chip and commercial devices across six distinct test subjects. The radar chart displays heart‐rate values (bpm) obtained from the proposed chip (red), Apple Watch Ultra 2 (blue), Huawei Watch GT4 (purple), and the medical‐grade Omron HPO‐100 pulse oximeter (yellow). The proposed chip exhibited excellent measurement consistency with the Omron reference device, maintaining a data discrepancy rate below 2%. Across all six subjects, the red contour (proposed chip) and yellow contour (Omron) overlapped almost completely. While the Apple and Huawei watches capture heart‐rate trends, the proposed device demonstrated superior conformity to the medical reference, which is particularly evident at the peak values observed in test subjects 1 and 3. This high‐fidelity alignment confirms that the fabricated chip achieves medical‐grade detection accuracy, validating its reliability for precision health monitoring applications.

Figure 5c compares the volume of the proposed integrated chip with those of three commercial optoelectronic modules (Product A,B, and C) obtained from disassembled mainstream smartwatches. A millimeter ruler serves as the size reference. Product A is a circular module with a 20‐mm diameter, Product B has a 15‐mm diameter, and Product C is further miniaturized to 10 mm. In contrast, the proposed chip features an ultra‐compact rectangular layout with dimensions of 5.4 mm × 3.1 mm. This volume comparison highlights the innovative nature of the embedded silicon integration method, which addresses the “size–function tradeoff” challenge in wearable biomedical electronic devices. By integrating LEDs, photodetectors, and digital ICs onto a single silicon wafer, the chip eliminates redundant packaging layers and interconnections found in commercial modules, significantly reducing the planar area. The chip area is approximately 94.7%, 90.5%, and 78.7% smaller than those of Products A (Huawei Watch GT2 Pro), B (Apple Watch S1), and C (Samsung Galaxy Gear S3), respectively. Importantly, this miniaturization does not compromise functionality. The ultra‐compact form factor enables “invisible” wearables, such as skin‐adhesive patches and ultrathin smart bracelets, avoiding the discomfort caused by bulkier commercial modules. Additionally, the reserved chip area allows future integration of complementary sensors, overcoming spatial limitations and enabling multiparameter physiological monitoring. This comparison demonstrates that the proposed integration strategy bridges the gap between microsystem prototypes and consumer‐grade wearable devices.

Figure 5d illustrates the improvement in chip static power consumption. The static power consumption of integrated systems comprises the power consumption of the chip and that of the interconnect structures during signal transmission. As this study primarily focused on integrated manufacturing processes, we concentrate on the static power consumption during signal transmission on the RDL. Commercial devices rely on wire bonding; however, as packaging technology advances toward ultrahigh‐density interconnects (pitch <200 µm, pad size <50 µm), wire bonding becomes inadequate owing to limitations in the wire curvature radius and pad contact area. In this study, we employed a semi fabricated copper RDL to form finer, high‐density interconnected structures on the chip surface. This method reduces the interconnect resistance by 43%, resulting in a 43% reduction in static power consumption. The improvement is primarily due to the RDL, which shortens the current paths, mitigates the high impedance of the wire bends, and improves energy efficiency. This low‐power interconnect solution offers an optimal approach for high‐density packaging and can be further optimized to satisfy multi‐chip integration demands.

Figure 5e compares our results with those of previous studies, with the horizontal axis indicating the chip type and the vertical axis indicating the corresponding area utilization. We define chips manufactured using different substrate materials (e.g., Si, GaAs, and SiC) and chip processes (e.g., process nodes, microelectromechanical systems (MEMS) processes, or IC processes) as distinct chip types. Area utilization was calculated as the ratio of the integrated sub‐chip area to the total chip area, serving as a metric for measuring the multi‐chip integration density on the interposer. As shown in the coordinate plot, the area utilization was <60% in most prior studies, failing to achieve high‐density integration of multiple chips. In contrast, benefiting from the layout algorithm, the proposed method achieved 90% area utilization owing to the high precision of silicon‐based processing (minimum spacing between embedded chips was <40 µm); this is significantly higher than those of other studies, highlighting its significant advantages in HI design. Moreover, in the integration of multiple chip types, the proposed method not only effectively enhanced the area utilization but also maintained excellent process compatibility, ensuring high performance and multifunctionality of the system. Thus, it significantly outperforms traditional design approaches in the integration of multiple chip types, offering a more efficient and compact solution. This advancement contributes to the development of multi‐chip integrated devices with substantial academic value and practical significance.

The proposed SEMHI methodology enables high‐density, multifunctional integration of chips with over five different substrate materials on a single interposer, effectively reconciles the intrinsic conflicts among device miniaturization, thermal management, and process compatibility. In contrast to conventional eSiFO approaches that mandate uniform chip thinning, SEMHI leverages MEMS multi‐depth etching technology to fabricate embedded cavities with varying depths. Consequently, photoresist distortion is eliminated during high‐aspect‐ratio etching, enabling the direct integration of chips with diverse thicknesses and significantly shortening R&D cycles. Critically, by incorporating B‐tree topology with heuristic optimization algorithms. SEMHI achieves system‐level thermal balancing, isolating temperature‐sensitive modules from heat‐generating sources while optimizing interconnect length and package volume. In terms of interconnect performance, SEMHI transcends the scalability limits of traditional microbump technologies (typically 20–80 µm) used in CoWoS and EMIB. By utilizing photolithography‐defined RDLs for direct connections, SEMHI achieves an ultrahigh input/output (I/O) density of 2.5*10^5^ mm^2^ and a minimal die‐to‐die spacing of 25 µm. which features spatial compactness and multi‐physics co‐optimization. Consequently, SEMHI provides a robust, high‐density solution for next‐generation, high‐bandwidth applications such as CPO, optical transceivers, and LiDAR.

## 4 Methods

### 4.1 Floorplan Algorithm

In system‐level floorplan optimization, all chips undergo comprehensive optimization at the individual chip level, with the placement and routing of standard cells fully completed. The entire floorplan optimization process is structured using the B*Tree floorplan generation algorithm as the basis for structural representation in conjunction with the simulated‐annealing algorithm to achieve optimization. The input parameters of the optimization process encompass three core categories of constraints. First, the inherent physical properties of the chips, such as dimensions and power consumption, are considered. Second, manufacturing process constraints are incorporated to ensure process feasibility; for example, the minimum spacing between any two chips is set to 20 µm, and this constraint is rigorously adhered to during the floorplan generation. Third, the simulation boundary conditions are defined, which serve as foundational parameters for the analysis of thermal characteristics. To achieve precise thermal floorplan optimization, it is essential to obtain the temperature‐field data corresponding to different floorplan configurations through simulations. In this study, COMSOL was employed for thermal simulation analysis, enabling the efficient output of steady‐state and transient temperature distribution data. Simultaneously, Python was used to implement the iterative solution of the optimization algorithm, creating a closed‐loop optimization process of “simulation–calculation–iteration.” This study comprehensively considered three key indicators: volume, wire length, and temperature, as given by Equation (2).

(2) $$F=\alpha \cdot Volume+\beta \cdot WL+\gamma \cdot T_{transient}+\varepsilon \cdot T_{stable}$$

here, α, β, γ, and ε are the weight coefficients for volume, wire length, transient temperature, and steady‐state temperature, respectively. Table 1 lists the input parameters adopted for the optimization. By adjusting their values, different optimization objectives—such as volume cost or temperature cost—can be prioritized. Importantly, the selection of temperature indicators considers the actual operating characteristics of the LED chips. The steady‐state temperature (*T_stable_*) refers to the maximum temperature at which all chips operate at a constant power, reflecting the heat‐accumulation effect during long‐term stable operation. The transient temperature (*T_transient_*) corresponds to the maximum temperature of the LED chip when driven by a square‐wave signal (with a period of 2 s and a duty cycle of 50%), which is more closely aligned with the thermal response characteristics under actual pulse operation. The optimization process adopts the simulated‐annealing algorithm. Its core mechanism is that during the iteration, if the cost value of the new floorplan is lower than that of the previous iteration, the new solution is directly accepted. If the cost value is higher, the acceptance of the new solution is determined by the Boltzmann probability function $exp((cost_{new}-\;cost_{last})/T)$, where *T* represents the current annealing temperature. This feature of “probabilistically accepting inferior solutions” can effectively prevent the algorithm from being trapped in local optima. The iteration terminates when the preset maximum number of iterations is reached or when the annealing temperature decreases to the minimum threshold *T_cold_*.

### 4.2 Integration Process

SEMHI was utilized to achieve tightly coupled integration of different chips. Figure 1a illustrates the specific process steps for multi‐chip integration based on this technology. Initially, embedding cavities with etching depth that precisely matches with the chip thickness was a critical requirement for ensuring surface uniformity. This process involved multiple photolithography and etching steps to fabricate embedding trenches of varying depths. Owing to chip thickness variations of several tens to hundreds of micrometers, traditional processes often result in uneven photoresist coatings on deep‐trench structures, leading to pattern distortion and accuracy loss. In the present study, we addressed this issue by designing a polymer–metal–silicon oxide multilayer etching process. As shown in Figure 3, on 4‐inch single‐polished silicon wafers, a 1 µm‐thick SiO_2_ layer was first grown at 250°C using the ULVAC CC‐200Cz system as the first mask layer. Subsequently, a 3 µm‐thick photoresist (AZ6130) was spin‐coated, patterned via exposure and development, and etched (NLD570) to transfer the pattern onto the SiO_2_. Next, a 500 nm thick aluminum film was evaporated using the ULVAC ei‐501z system. The photolithography and etching steps were repeated, and Al was etched in a chlorine atmosphere using Naura GSE C200 equipment to form the second layer pattern. A 7 µm‐thick photoresist (AZP4620) was spin‐coated as the third mask layer. Al and SiO_2_ were sequentially etched to obtain the composite pattern. After pattern formation, three‐step deep silicon etching was performed using an SPTS Omega LPX system: the second etching step reached a depth of 75 µm and removed the entire Al mask; finally, the third etching step reached a depth of 65 µm to achieve a multi‐level stepped trench structure. This process effectively mitigates the negative impact of deep trench structures on lithographic alignment and linewidth control by optimizing the SiO_2_/Al/thick photoresist composite mask system and the layered removal mechanism, thereby enhancing overall pattern accuracy and structural uniformity.

Owing to the variations in the electrode distribution across different chip types, some chips feature electrodes on both the top and bottom surfaces. In such cases, the corresponding bottom electrodes are fabricated at the bottom of the embedding cavities using a semifinished copper process. As shown in Figure 3d, after etching completion, a 1 µm thick SiO_2_ insulating layer was deposited on the embedded cavity surface and sidewalls using the ULVAC CC‐200Cz system at 250°C. Subsequently, a 100 nm thick Ti bonding layer and 1000 nm thick Cu seed layer were sequentially grown using the YNS 800 magnetron sputtering system. To ensure uniform copper coverage within the deep cavity, the seed‐layer thickness was increased slightly beyond standard process specifications. Subsequently, a 10 µm thick layer of photoresist (AZP4620) was spin‐coated onto the wafer surface. After exposure and development to define the electroplating window, a 5 µm thick Cu layer was electroplated using a deep‐hole spray plating system (DT‐MKPD‐1). Following electroplating, the exposed seed layer was removed using an ion‐beam etcher (IBE‐200) to achieve metallization within the embedded cavities. Prior to embedding, a Die attach Film(DAF) layer was pre‐coated on the backside of each chip to enhance adhesion with the embedding trenches. The thickness and conductive properties of the DAF were optimized according to device requirements. Pre‐treated chips were placed a submicron placement machine (HXWZ‐Hx10A), pressed into the trenches at 150°C and 2‐N pressure for 1 min, and then cured at 180°C in a nitrogen atmosphere for 2 h to enhance the bonding strength. Following wafer re‐bonding, a vacuum lamination process was performed: a 25 µm‐thick photosensitive dry film (TOK S2020) was applied to the wafer surface at 75°C, 5 kg pressure, and 1 Torr vacuum. This film filled the grooves between the embedded chips and the interposer layer while achieving surface planarization. To enable chip interconnections, the photoresist was exposed to 800 mJ of energy to reveal pad areas. Subsequently, 100 nm of Ti and 1000 nm of Al were deposited via magnetron sputtering. Dry etching (GSE C200 system) using Cl_2_ and BCl_3_ gases formed the metal interconnect patterns, completing the integrated‐chip assembly.

## Funding

This work was supported by the National Key R&D Program of China (Grant No. 2023YFB4404100), the National Natural Science Foundation of China (Grant No. 52521007), and the China Postdoctoral Science Foundation (Grant No. 2025M770514).

## Ethics Statement

This study was approved by the Ethics Committee of Peking University Biomedical Ethics Committee(Approval No. IRB00001052‐25165). Written informed consent was obtained from all participants. The study complied with the Declaration of Helsinki.

## Conflicts of Interest

The authors declare no conflicts of interest.

## Supporting information




**Supporting File**: advs75416‐sup‐0001‐SuppMat.docx.

## Data Availability

All data generated or analyzed during this study are included in this published article and its supplementary information files.
