# Supplementary material for: Silicon‐Embedded Multifunctional Heterogeneous Integration for Miniaturized Photoplethysmography Detection Devices
Source: Adv Sci (Weinh). 2026 Apr 28;13(40):e75416. doi: 10.1002/advs.75416 (PMC13335505; doi:10.1002/advs.75416)
Supplement: Supplementary file 1 — Supporting File: advs75416‐sup‐0001‐SuppMat.docx. [file ADVS-13-e75416-s001.docx]

**Thermal Testing System and Test Method**

In this experiment, infrared imaging is employed to achieve the visual measurement of chip temperature, with its theoretical basis being Planck's blackbody radiation law: the infrared radiation intensity of an object's surface has a quantitative correspondence with temperature:

$$M_{\lambda}\left( T \right)=\frac{2\pi hc^{2}}{\lambda^{5}}\cdot\frac{1}{e^{\frac{hc}{\lambda kT}}-1}$$

$M_{\lambda}\left( T \right)$ refers to the spectral radiance at a wavelength of λ (W·m⁻²·μm⁻¹), where h is Planck's constant, c is the speed of light, k is Boltzmann's constant, and T is the absolute temperature. The infrared imager collects infrared radiation signals from the chip surface, and by combining the Planck's blackbody radiation law with the correction of the chip surface emissivity, converts the radiation intensity into a temperature distribution image, thereby achieving real-time monitoring of the chip temperature.


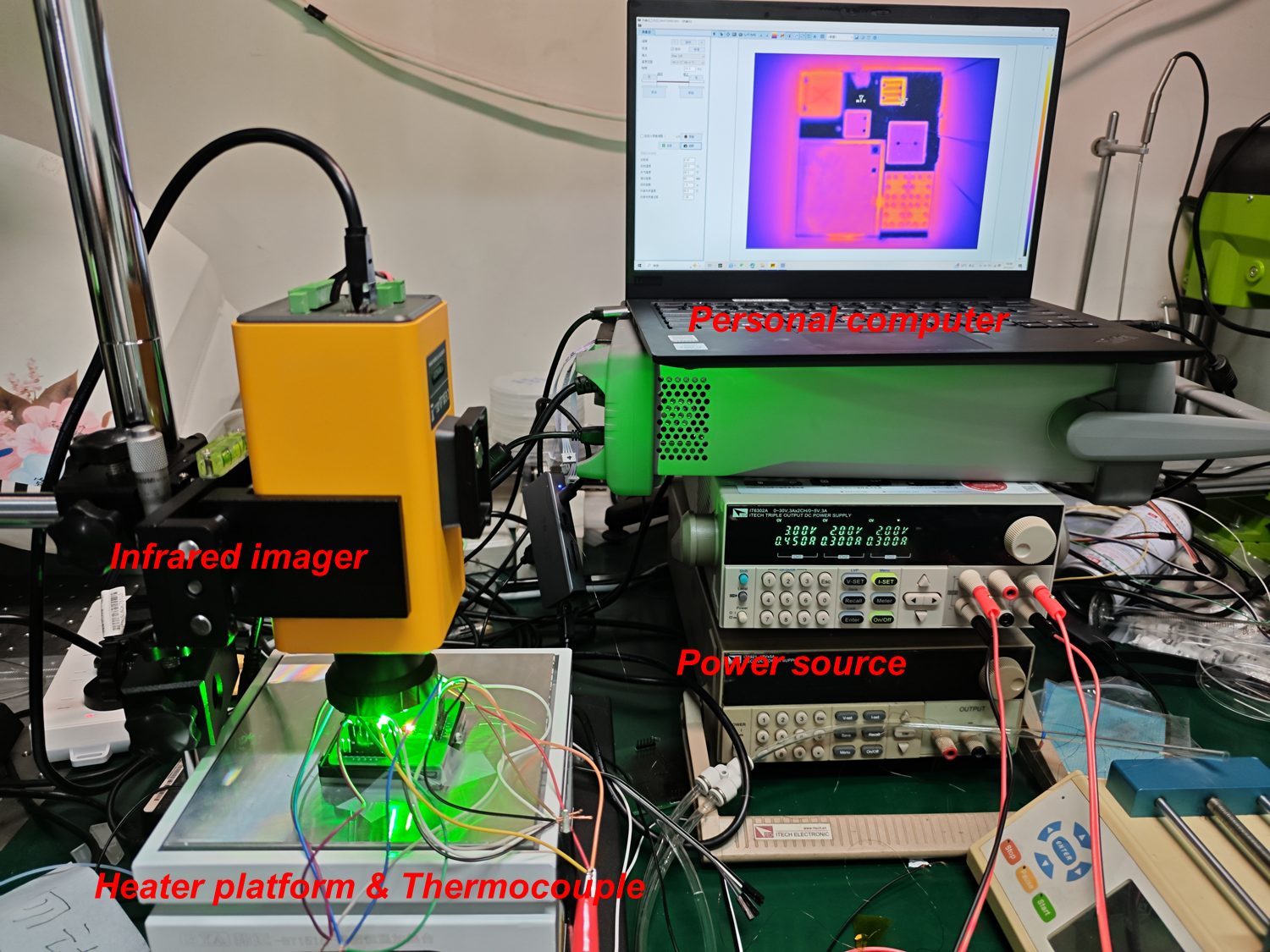


Figure S1:Thermal Test Experimental Platform

Figure S1 presents the established test platform for chip temperature measurement in this study, including infrared imager, heater platform, thermocouple, DC power and personal computer. The infrared imager collects infrared radiation from the chip and converts it into a temperature image. The heater platform can provide a stable temperature environment for emissivity calibration. The thermocouple serves as a temperature standard to calibrate the readings of the infrared imager. The DC power provides a stable rated operating voltage for the chip. Personal computer runs temperature measurement software (FLIR Research IR) to realize data storage and analysis. All devices are linked through standardized interfaces to ensure temperature measurement accuracy, and the key parameters of the core devices are listed in Table S1:

| Table S1: Parameters of the devices | | |
| --- | --- | --- |
| Experimental tools | Device model | Parameters |
| Infrared imager | FLUKE-RSE60 | Resolution:640×480;  Phase spacing:11μm;  Thermal sensitivity:30mK@30°C;  Accuracy:±2%@35°C;  Full-scale:-20~650°C. |
| Heater platform | BY1515 | Heating plate: 150mm×150mm;  Full-scale:0~400°C;  Power:550W. |
| Thermocouple (T-type) | KAIPUSEN | Accuracy:±0.3°C;  Full-scale:-200~200°C. |
| Power source | SPV SS-6 | Full-scale:0~30V; 0~5A;  Accuracy:0.03%±10mV@30°C. |
| Personal computer | ThinkPad X1 | - |

The typical working temperature range for the chip is 20–60°C. Therefore, calibration experiments are conducted within this range, with the core calibration parameter being the chip surface emissivity ($\varepsilon$), which directly influences infrared radiation measurement deviations as it varies with temperature. Real-time correction is required through a thermocouple. The calibration process begins with sample fixation, where the chip is placed at the center of a heated constant temperature platform, ensuring that the bottom surface of the chip is in close contact with the platform (thermal conductive grease is applied to reduce thermal resistance). Simultaneously, a T-type thermocouple probe is tightly affixed to the backside of the chip’s core heating region using high-temperature tape, ensuring there is no air gap between the probe and the chip. Next, the temperature gradient is set by the platform control system, adjusting the temperature from 20°C to 60°C in 2°C increments, with a 30-minute thermal equilibrium time after each 2°C increase (waiting until the thermocouple reading fluctuation is ≤0.1°C/5min) to ensure the chip temperature stability. Emissivity calibration is then performed at each stable temperature point. The infrared imager is activated and aligned with the chip’s core region. The emissivity parameter is gradually adjusted in the software until the average temperature of the core region displayed by the infrared imager deviates by ≤±0.1°C from the thermocouple-measured temperature. The emissivity value corresponding to this temperature point is recorded to form the “Temperature - Emissivity” calibration curve. Finally, reproducibility is verified by performing three repeat calibrations at the intermediate temperatures of 30°C, 40°C, and 50°C. The average emissivity value and standard deviation are calculated to validate the reliability of the calibration results.

The infrared system calibration and optimization begin with adjusting the focal length and field of view. The focal length of the infrared imager is adjusted to ensure clear surface texture on the chip, while the imaging angle is modified by translating the stage to ensure that the imaging plane is perpendicular to the chip's surface, thus avoiding radiation intensity loss due to the cosine effect. Following this, software parameters are configured. The temperature measurement software on the PC is set to a data collection frequency of 1 Hz, with the image storage resolution consistent with the imager's resolution (640×480 pixels). The data storage formats are CSV for temperature data and TIFF for raw infrared images to facilitate subsequent analysis.

Before temperature measurements, environmental preprocessing is performed. The experimental system is placed in a constant temperature environment, with the ambient temperature controlled at 20±1°C. Data collection begins with chip activation by applying the rated operating voltage using a DC stabilized power supply. Dynamic data collection is then carried out, with the data collection duration set to 30 minutes. The software automatically records temperature and image data at 1-minute intervals. To ensure the accuracy and reliability of the measurements, the entire temperature measurement and data collection process must be conducted in a stable environment to prevent external factors from interfering with the results.

**RDL Signal Transmission Characteristics of SEMHI**

The key distinction between SEMHI and other integration methods lies in the reconstruction of signal transmission pathways between different chips on the surface after embedding the chip into the silicon adapter plate. To evaluate the impact of the embedded structure and surface topography on signal transmission, we annotated critical parameters of the embedded structure as shown in Figure S2(a). Additionally, we established the transmission line test equipment and probe testing process depicted in Figure S2(b). The test system consists of two parts: the first is a vector network analyzer, which generates an RF signal of known frequency and amplitude through the transmission line under test. At this point, a portion of the signal may reflect back at the input or output end of the transmission line. The vector network analyzer measures the amplitude and phase of these reflected signals to obtain S11 (input reflection coefficient) and S22 (output reflection coefficient). Simultaneously, the instrument also measures the amplitude and phase of the signal propagating through the transmission line. These measurements can be used to determine S21 (forward transmission coefficient) and S12 (backward transmission coefficient). The second component of the test system includes a multi-magnification microscope for locating the sample position, magnifying the transmission line pad for needle insertion, and a pair of Cascade GSG high-frequency probes for transmitting the input/output signals to the vector network analyzer. This test system enables the measurement of S-parameters for signal transmission lines under varying structural and material parameters.

*
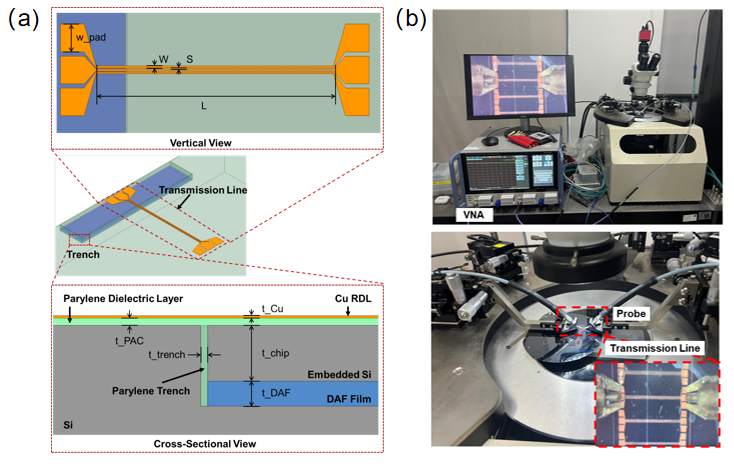
*

Figure S2: Parameter Specifications and Test Environment with Test Equipment. (a) GSG test structure and key parameter specifications; (b) Test probe station and chip testing process.

The primary structural difference between embedded fan-out and conventional silicon interposer lies in the inclusion of a trench between the embedded die and the interposer board, coupled with a certain height difference between them. Therefore, we primarily investigated the impact of altering these two parameters on signal transmission characteristics. The S-parameter loss at different slot widths is shown in Figure S3(a). As seen in the figure, since the slot width accounts for a very small portion of the entire transmission line structure, it has little significant impact on S-parameter loss. Due to the difficulty in precisely controlling chip thinning errors and trench etching errors, the height difference between the embedded chip and the silicon adapter plate was controlled during actual sample fabrication by adjusting the etching depth of the chip embedding trench. A trench depth 5 µm less than the chip thickness was defined as the chip being above the silicon adapter plate, while the opposite was defined as the chip being below the silicon adapter plate. Figure S3 (b) illustrates the effect of the height difference between the chip and the silicon adapter plate on high-frequency signal transmission loss. As shown in the figure, this height difference has almost no effect on the S-parameters.

*
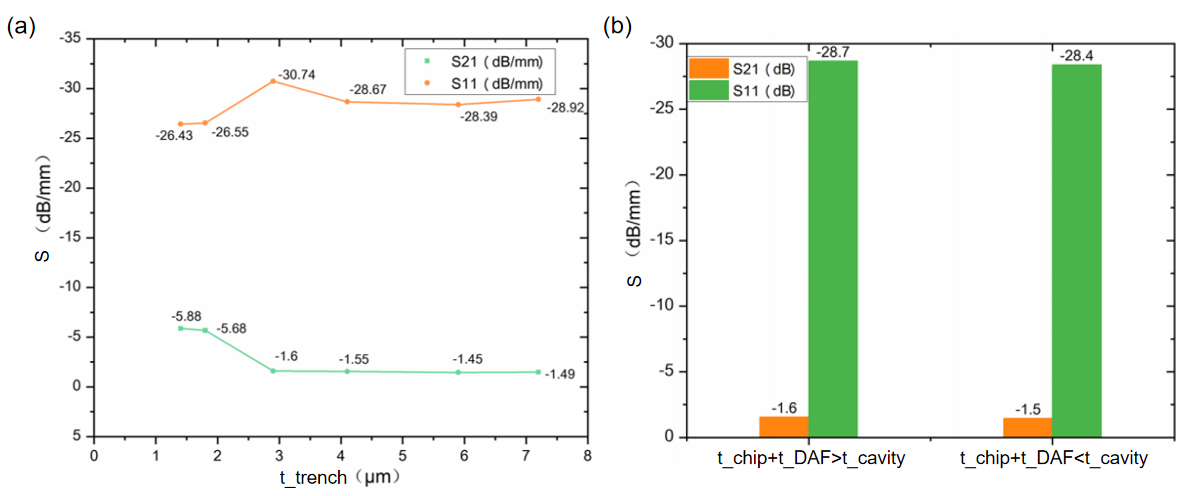
*

Figure S3: Impact of Different Trench Widths and Chip Contour on Signal Transmission. (a) Effect of trench width on signal transmission loss; (b) Effect of chip height difference on signal transmission loss.

Finally, we also compared the transmission line characteristics of embedded fan-out structures with those on silicon interposer. Their S-parameter characteristics at different frequencies are shown in the figure S4 below. The GCPW, incorporating a ground plane, provides superior electromagnetic shielding. This reduces interference from external noise and internal signal radiation loss, thereby enhancing signal transmission efficiency. Consequently, it exhibits lower insertion loss and better transmission characteristics across all three sample sets. For the CPW routing on the standard adapter board and the CPW routing on the embedded silicon fanout, the differences were minimal. In certain frequency bands, the embedded fanout even outperformed the standard silicon interposer. This is likely because the embedded process reduces signal reflections to some extent, thereby improving signal transmission efficiency.

*
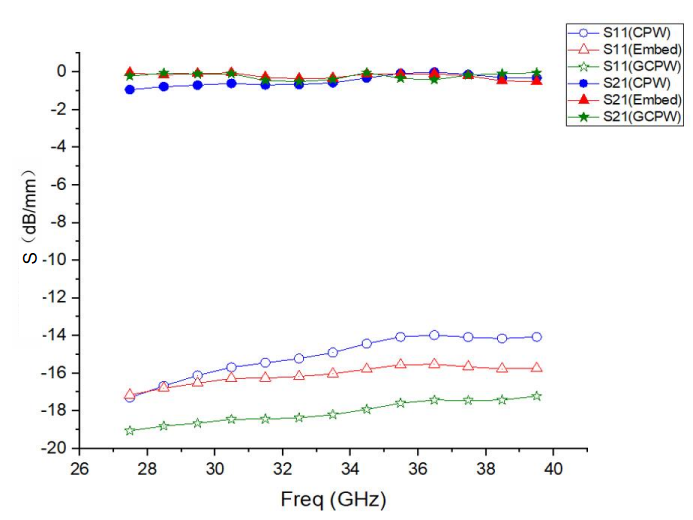
*

Figure S4 : Comparison of signal transmission characteristics between RDLs on embedded fan-out and conventional silicon interposer.

**Integrated Chip Information**

The detailed Parameters of the chips used for integration in this work are shown in Table S2.

Table S2: Parameters of the integrated chips

| Chip Model | Material | Size(μm) | Manufacturer | Function |
| --- | --- | --- | --- | --- |
| EK-26B | GaN Epitaxial on SiC | 640*640*160 | San'an Optoelectronics | Green Light Emission |
| M26ARS6UA | AlGaInP Epitaxial on Si | 660*660*180 | Qianzhao Optoelectronics | Red Light Emission |
| BN-I4242AC | GaN Epitaxial on Sapphire | 1060*1060*200 | Epileds Technologies | Infrared Light Emission |
| A100-3010PD | Si Device | 2500*2600*120 | Xilan Optoelectronics | Photoelectric Sensing |
| GC6130A | Si IC | 1450*1600*65 | Beijing Shuangjing Integration Co., Ltd. | Signal Processing |

**Impact of Embedding Process on Chip Performance**

To verify the impact of the embedded process on the digital IC amplifier, this paper designed the on-chip test circuit schematic shown in Figure S5(a). The operational amplifier operates under deep negative feedback conditions. Based on the “virtual short” principle of an ideal operational amplifier, the voltages at the VOUT and VIN ports should be identical at this point, thereby producing a voltage-following effect. By testing the voltage-following capability of the operational amplifier, the normal operation of this CMOS chip is verified. A chip-on-board test system was constructed by connecting a semiconductor parameter analyzer based on the 4156B model with a Karlheinz PM8 probe station, as shown in Figure S5(b). Probe a and probe b contact the chip's positive and negative power supply ports, respectively. Probes c and e contact the chip's non-inverting and inverting input ports, respectively. Probe d contacts the chip's output port. A three-way connector links probes d and e to implement the operational amplifier's negative feedback. The semiconductor parameter analyzer's four channels are configured as the VDD, VSS, VIN, and VOUT SMU ports. The VDD port outputs a constant 3 V supply voltage and is connected to probe a. The VSS port outputs a COMMON ground signal and is connected to probe b. The VIN port outputs a ramp voltage signal ranging from 0.5 V to 2 V and is connected to probe c to measure the operational amplifier's input voltage. The VOUT port outputs a constant 0 V voltage and is connected to probe d to measure the operational amplifier's output voltage. By comparing the voltage signals acquired at the VIN and VOUT ports, the chip's voltage-following capability can be verified.

Figure S5(c) and (d) show the voltage tracking performance on-chip after die bonding to the silicon substrate and after die pad windowing, respectively. The maximum relative deviation in voltage tracking after die bonding to the silicon substrate was 5%, with an average absolute deviation of 2.1 mV, indicating excellent voltage tracking performance. This confirms that the dicing, thinning, and bonding processes did not damage the die. After die pad windowing, the maximum relative deviation in voltage tracking was 4%, with an average absolute deviation of 1.3 mV. The chip demonstrated good voltage tracking capability, confirming that processes such as planarization and polymer etching did not adversely affect the chip. The on-chip test results validate that the buried CMOS chip functions normally, indicating good process compatibility.


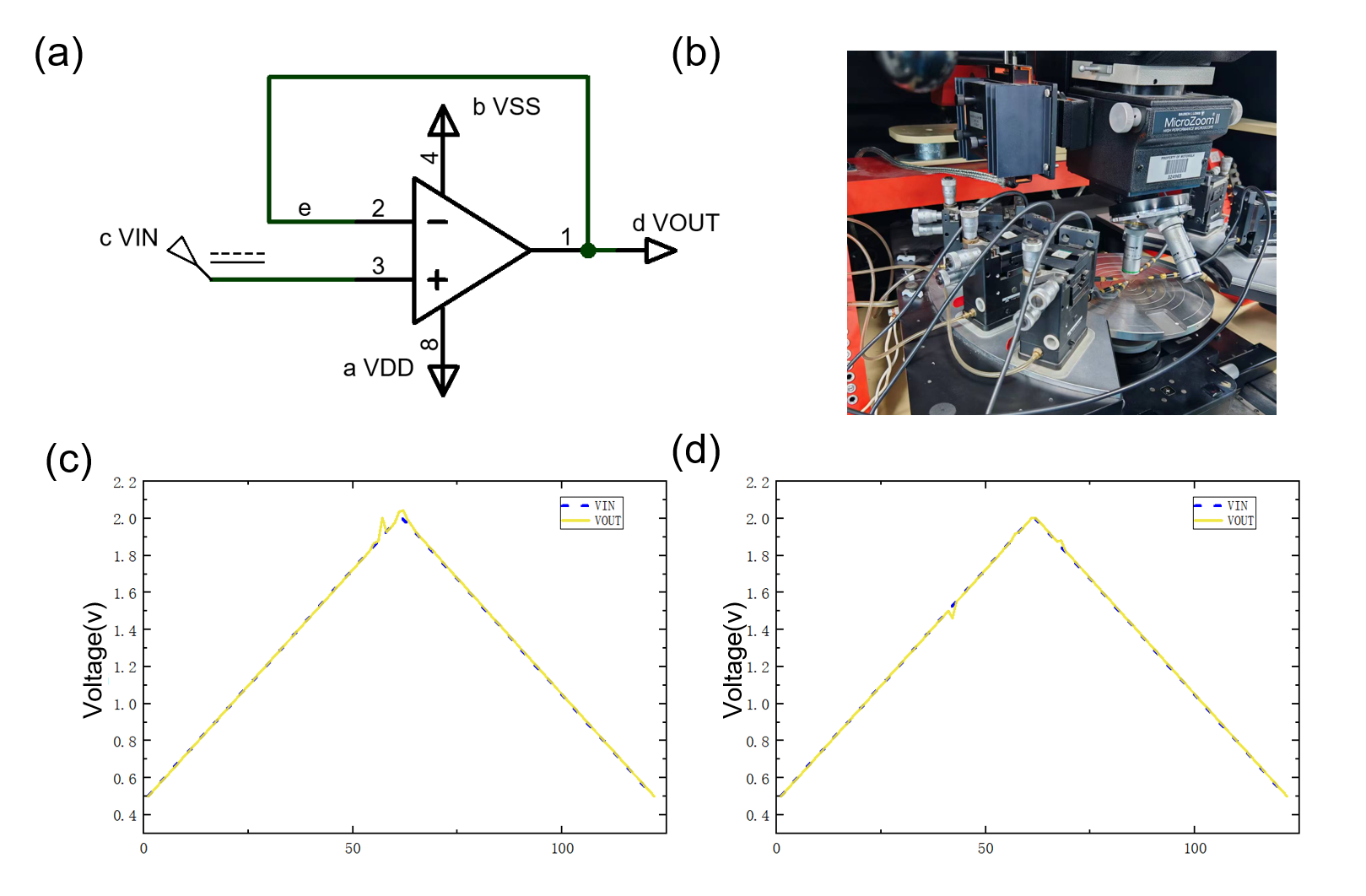
Figure S5: On-chip Performance Testing Method and Results for Digital IC Chips During the Embedding Process. (a) On-chip test circuit; (b) Probe station test equipment; (c) Performance test results after chip embedding in silicon interposer; (d) Performance test results after chip pad windowing.

Additionally, for fully encapsulated chips, we designed a signal inverting amplifier circuit as shown in Figure S6(a) to perform single-function verification and evaluation. A PCB board as depicted in Figure S6(b) was fabricated for peripheral signal processing. The results are illustrated in Figures S6(c) and (d). A signal generator provided square and sine wave excitations with a peak-to-peak amplitude of 100 mV to the Vin port. A data acquisition card captured the output waveform from the operational amplifier's Vout port. The captured waveform exhibited a peak-to-peak amplitude approaching 1 V with a relatively complete waveform pattern. These results confirm that the encapsulated chip operates effectively, demonstrating that the embedding process has negligible impact on the performance of the digital IC chip.


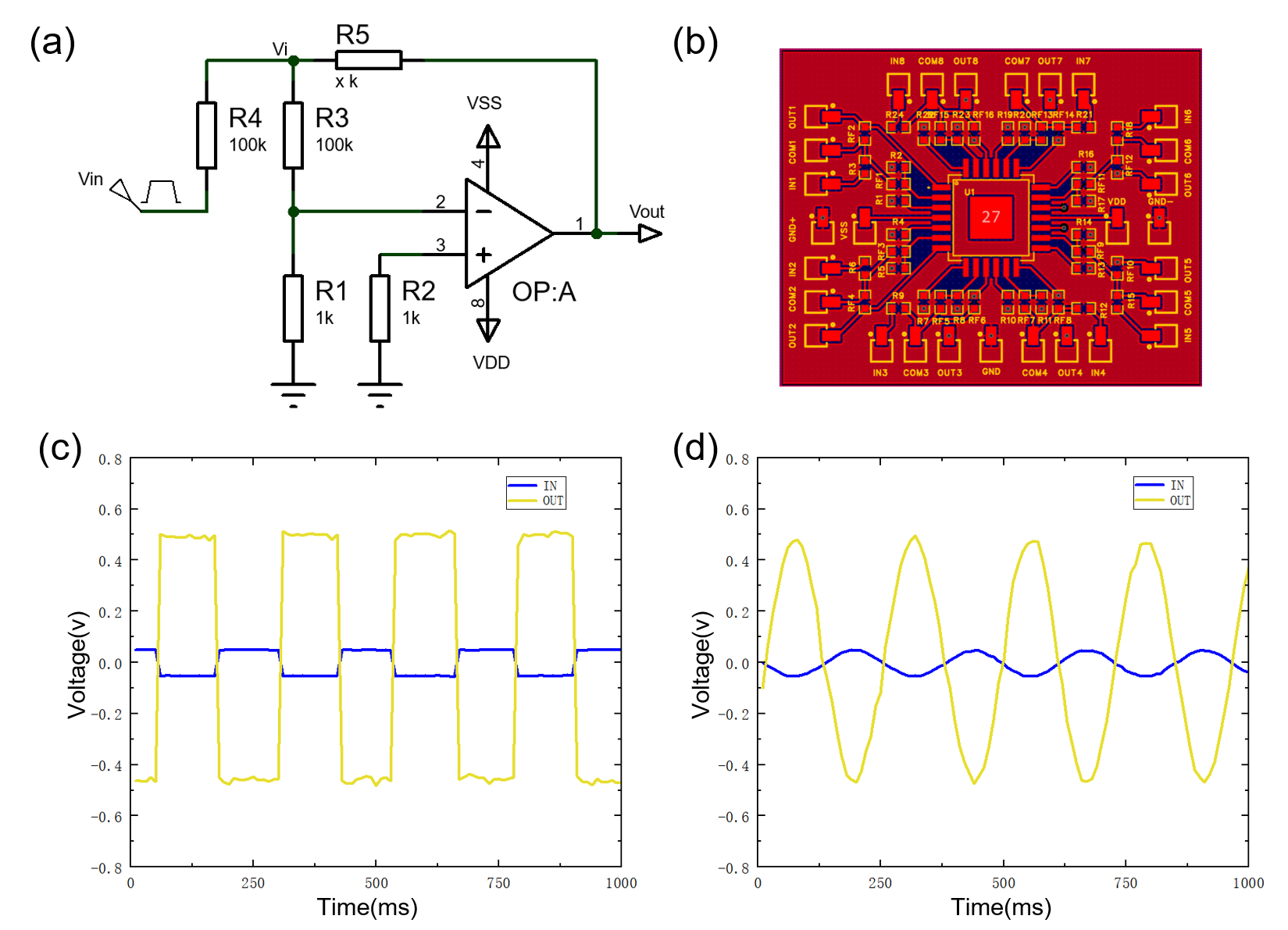


Figure S6: Board-level testing of chip performance. (a) Schematic design of the encapsulated chip test circuit; (b) PCB layout; (c) Inverted tracking effect of the encapsulated chip on a square wave; (d) Inverted tracking effect of the encapsulated chip on a sine wave.

Finally, we also conducted individual functional tests on the optoelectronic sensors after encapsulation to characterize whether their performance was affected by the embedding process. Figure S7(a) shows the BF1500 probe station used, which features light-shielding capabilities making it more suitable for sensor testing. We measured the response IV curves under pure darkness, natural light, and 800 nm laser illumination. Results in Figure S7(b-c) indicate that the current-voltage (IV) characteristics of the sensor under different environmental conditions exhibit no significant changes, whether before or after encapsulation. Figure S7(d) shows that the fluctuations in the two curves primarily stem from the use of a handheld laser during testing. The inconsistent positioning of the laser beam during measurements caused minor deviations in the curves. Based on the above analysis, it can be concluded that the SEMHI packaging process did not significantly impact the performance of this photodetector, or any effects observed remain within an acceptable range.


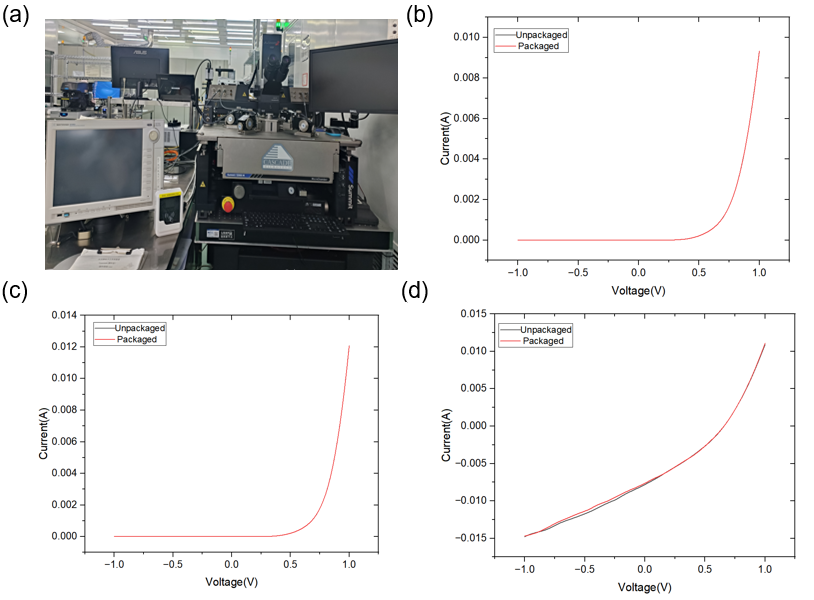


Figure S7: Performance Testing of Photoelectric Sensors During Pre- and Post-SEMHI Packaging. (a) PD functional test probe station; (b) IV characteristic testing of PD under dark conditions; (c) IV characteristic testing of PD under visible light irradiation; (d) IV characteristic testing of PD under 800 nm laser irradiation.
